# Missing a “Missing Self” Mechanism: Modeling and Detection of Ly49 Expression in Canine NK Cells

**DOI:** 10.4049/immunohorizons.2300092

**Published:** 2023-11-16

**Authors:** Alicia A. Gingrich, Aryana M. Razmara, Phillip W. Gingrich, Robert B. Rebhun, William J. Murphy, Michael S. Kent, C. Titus Brown, Justin B. Siegel, Robert J. Canter

**Affiliations:** * Department of Surgery, University of California, Davis School of Medicine, Sacramento, CA; † Department of Biochemistry and Molecular Medicine, University of California, Davis School of Medicine, Sacramento, CA; ‡ Department of Surgical and Radiological Sciences, University of California, Davis School of Veterinary Medicine, Davis, CA; § Department of Dermatology, University of California, Davis School of Medicine, Sacramento, CA; ¶ Department of Population Health and Reproduction, University of California, Davis School of Veterinary Medicine, Davis, CA

## Abstract

NK cells are a key focus in immuno-oncology, based on their ability to eliminate malignant cells without prior sensitization. Dogs are valuable models for translational immunotherapy studies, especially for NK cells, where critical species differences exist between mice and humans. Given that the mechanism for recognition of “self” by canine NK cells is currently unknown, we sought to evaluate expression of Ly49 in canine NK cells using in silico and high-throughput techniques. We interrogated the identified polymorphism/mutation in canine Ly49 and assessed the potential impact on structure using computational modeling of three-dimensional protein structure and protein-protein docking of canine Ly49 with MHC class I (MHC-I). Bulk and single-cell RNA-sequencing analysis was performed to detect gene expression of Ly49/KLRA1 in resting and activated NK cells. Tertiary protein structure demonstrated significant structural similarity to the known murine system. Molecular docking of canine Ly49 with MHC-I was favorable, converging at a single low-energy conformation. RNA sequencing revealed expression of Ly49/KLRA1 in both resting and activated NK cells and demonstrated almost exclusive expression of the gene in the NK cluster at the single-cell level. Despite prior reports of a mutated, nonfunctional canine Ly49, our data support that the protein product is predicted to bind to MHC-I in a comparable conformation to the murine system and is expressed in canine NK cells with upregulation following activation. Taken together, these data suggest that Ly49 is capable of recognizing MHC-I and therefore regulating NK cell function in dogs.

## Introduction

NK cells are innate lymphoid cells that possess the ability to kill virally infected and malignantly transformed cells without prior Ag sensitization. For this reason, NK cells have become a focus of research to harness immunotherapy for the treatment of cancer, particularly in tumors where the response to T-cell–based therapies has been modest (1–3). Experiments in mouse models have been invaluable to understanding mechanistic concepts of tumor immunology and immunotherapy, including NK cell function and response (1, 4, 5). However, intrinsic characteristics of mouse models create challenges for translational applications of NK immunotherapy to clinical practice, because inbred mice demonstrate key differences in NK immunobiology (6).

Comparative oncology has leveraged the canine model, which has emerged as a relevant model for studying cancer biology and immunotherapy (7–9). Like humans, dogs are large, outbred mammals that develop spontaneous cancers as they age in the setting of an intact immune system. Previous studies on canine cancers have demonstrated shared genetic driver mutations and tumor biology with human cancers, such as osteosarcoma, lymphoma, gliomas, and melanoma (10–13). Given the importance of the interplay between the genomic alterations of tumors and the associated immune response, few species are as well suited for translational immunotherapy studies as companion canines.

Although NK cells have been studied extensively in humans and mice, detailed studies are lacking in dogs, and, as a result, canine NK cells are relatively poorly defined (14, 15). Notably, human and mouse NK cells have inhibitory receptors that inhibit NK responses to cells bearing the MHC class I (MHC-I) marker. This is known as the “missing self” hypothesis, in which NK cell stimulation by activating receptors is prevented in the presence of MHC-I/inhibitory receptor binding (1, 16). Human NK cells have a group of pleomorphic inhibitory MHC-I–specific receptors known as killer Ig receptor (KIRs), whereas murine NK cells have lectin-like Ly49 dimers as their surface MHC-I binding receptor (17). A third MHC-I inhibitory receptor, the CD94-NKG2A heterodimer, is present in both species (17, 18). Key steps in NK cell regulation occur, based on the integration of the activating and inhibitory signal inputs from such receptors (1, 19).

However, the mechanism for which canine NK cells recognize and bind MHC-I for the purpose of NK cell inhibition is currently unknown. It is thought that the KIR and Ly49 genes have undergone convergent evolution (20). The KIR gene family is expanded in humans and has overridden the Ly49 family, which exists as a single pseudogene in humans (17, 21). In mice, Ly49 has expanded to be a multigene family (17, 22). Analysis of the dog genome for evidence of Ly49 and KIR genes has been studied previously (21, 23, 24). Using Southern blot techniques, Gagnier et al. determined that dogs have one copy of the Ly49 gene, which contains an ITIM domain (21). However, it was noted that the dog Ly49 gene has undergone a cysteine-to-tyrosine conversion at position 168, which is one of six highly conserved cysteine residues in the C-type lectin-like domain that is involved in disulfide bonds (25). Without this cysteine, it was reasoned that the capability of the dog Ly49 gene to function is unclear, prompting some authors to hypothesize that the dog Ly49 gene is nonfunctional (21). Subsequently, Hammond et al. studied the KIR gene family in mammals (24). Based on genome build 2.1 assembly for dogs (current assembly is genome build 4.1), the KIR gene appeared to be absent, with nearby genes truncated (24). Interpreting this evidence, the authors concluded that dogs also lack a functional KIR gene (24).

Although each of these studies was well designed and evidence based, taken together, the logical conclusion is that dog NK cells lack a reliable mechanism to recognize MHC-I, given the mutated Ly49 gene and the absent/truncated KIR gene. Because MHC-I recognition is critical to NK self-tolerance across species, we reasoned that the implications of these prior studies left a gap in the literature. Therefore, we sought to evaluate the structure and expression of Ly49 in canine NK cells using novel techniques, which may shed additional light on the potential consequences of the cysteine-to-tyrosine mutation in the Ly49 gene. Because the field remains limited by lack of species-specific Abs for canines, we used in silico protein structure modeling and protein-protein docking to estimate the structure of Ly49 and its complex with MHC-I and performed high-throughput sequencing to test for gene expression. We hypothesized that, despite the cysteine-to-tyrosine modification, Ly49 is structurally preserved in canines and is a candidate for inhibitory NK cell regulation with MHC-I.

## Materials and Methods

### Molecular modeling

The canine DLA-88 MHC-I Ag has been characterized crystallographically in Protein Data Bank entry 5F1N (26). For modeling, waters were removed in PyMol 2.4, and chains A, B, and C were retained as input for protein-protein docking. AlphaFold2 (27) was used to generate structural models of canine Ly49, specifically for the extracellular portion of the sequence. This was performed using ColabFold (28). Summarily, multiple sequence alignments were generated automatically using MMseqs2 within ColabFold (29). Models were generated without the use of templates to avoid biasing the output structures to the known Ly49 structures within the Protein Data Bank. Three recycles of each model were performed for a total of five models. Each model was submitted to a constrained relaxation using the OpenMM 7 (30) to alleviate clashes within the generated models. The top-ranked structure was compared with chain D from 1P1Z (31) to make a comparison with an experimentally solved murine structure.

Protein-protein docking was performed using ensemble docking within Rosetta 3.12 (31). Structural ensembles for both the DLA-88 complex from the 5F1N structure and the AlphaFold-generated Ly49 models were generated to better explore conformational space during docking due to the rigid body treatment of protein-protein docking in Rosetta. Each protein structure was subjected to a single constrained relaxation to alleviate small clashes while largely conserving the input structure, and then five unconstrained relaxations of each input were performed to explore structural space farther away from the original input structures. This resulted in 7 conformations for the DLA-88 ensemble and 35 conformations for the Ly49 ensemble.

From the Protein Data Bank, murine MHC-I in complex with Ly49C (entry 1P1Z [32]) was taken for comparison with generated complex structures. The initial input structure containing the canine DLA-88 complex and top-ranked AlphaFold model of Ly49 was aligned to the murine structure in PyMol. This initial geometry was used as a reference point for root mean squared deviation calculations for comparison of generated structures. Rigid body docked structures were generated by ensemble docking. Convergence was examined on the basis of the protein-protein interface score in Rosetta Energy Units and the root mean squared deviation as compared with the aligned input structure measured in angstroms (Å).

### Processing of canine NK cells

For bulk RNA sequencing, canine whole blood was obtained from healthy male and female beagles (Ridglan Farms, Mount Horeb, WI) aged 2–8 y old. Blood from a tumor-bearing canine patient with melanoma enrolled on an institutional animal care and use committee– and clinical trials review board–approved clinical trial (protocol 21620) prior to treatment was obtained for single-cell RNA sequencing (scRNA-seq). As a healthy comparison, blood from a female 7-y-old beagle was also obtained for scRNA-seq. PBMCs were collected using a density gradient (Lymphocyte Separation Medium, Corning Life Sciences), as described previously (33, 34). RBCs were eliminated by incubation with RBC lysis buffer for 5 min at 4°C. Cells were then washed with PBS and used for subset isolation. A subset of beagle PBMCs underwent magnetic bead depletion of CD5^bright^ cells to enrich for of CD5^dim^ cells using the Easy Sep PE Positive Selection Kit (STEMCELL Technologies, Vancouver, BC, Canada) and PE-conjugated anti-canine CD5 (Invitrogen, clone YKIX322.3).

### Cell sorting

Beagle PBMCs were washed with PBS and incubated with Fc receptor blocking solution (Canine Fc Receptor Binding Inhibitor, Invitrogen, catalog no. 14-9162-42), then stained with CD3-FITC (clone CA17.2A12, Bio-Rad Laboratories, catalog no. MCA1774F), NKp46-PE (clone 48A, kind gift of Dr. Dean Lee), and live/dead staining performed using Fixable Viability Dye 780 (eBioscience, catalog no. 65-0865-14). Stained cells underwent flow cytometric analysis and sorting of CD3-NKp46^+^ live cells using a Beckman Coulter Astrios EQ 18 color cell sorter. Resulting cells were sorted directly into 96-well plates with QIAseq lysis buffer and RNase inhibitor.

### Ex vivo NK cell activation and expansion of canine NK cells

PBMCs, CD3-NKp46^+^ sorted, and CD5-depleted cells from beagle donors were cocultured with irradiated K562 human feeder cells transduced with 4-1BBL (CD137L) and membrane-bound rh-IL21 (K562C9IL21, courtesy of Dr. Dean Lee, Nationwide Children’s Hospital, Columbus, OH) at a ratio of 1:2 (NK/feeder cells) supplemented with 100 IU/ml rh-IL2 (35–38). The parental K562 cell line was originally obtained from American Type Culture Collection prior to engineering of transgene expression (33). rhIL-2 (100 IU/ml; National Cancer Institute, Frederick, MD) was added every 2–3 d while NK cells were in culture. Every 7 d, cells were counted and resuspended at a concentration of 250,000 NK cells/ml with fresh K562C9IL21 at a ratio of 1:1, as previously described (33, 38). A subset of resting CD5-depleted cells was activated by exposure to 100 ng/ml rhIL-15 for 3 d. Cells were tested approximately every 6 mo to authenticate (by short tandem analysis) and to confirm *Mycoplasma* negativity.

### Bulk sequencing and scRNA-seq

RNA from beagle PBMCs and CD5-depleted cells before and after 14-d coculture was extracted using RNeasy Mini kits (Qiagen). Total RNA was submitted to the University of California (UC) Davis Genome Center for quality assessment, library preparation, and sequencing. Gene expression profiling was carried out using a 3′-Tag-RNA-Seq protocol. Barcoded sequencing libraries were prepared using the QuantSeq FWD kit (Lexogen, Vienna, Austria) for multiplexed sequencing according to the recommendations of the manufacturer using both the unique dual-indexed adapter and unique molecular identifier second-strand synthesis modules (Lexogen). The fragment size distribution of the libraries was verified via microcapillary gel electrophoresis on a LabChip GX system (PerkinElmer, Waltham, MA). The libraries were quantified by fluorometry on a Qubit fluorometer (Life Technologies, Carlsbad, CA) and pooled in equimolar ratios. The library pool was quantified via quantitative PCR with a Kapa Library Quant kit (Kapa Biosystems/Roche, Basel, Switzerland) on a QuantStudio 5 system (Applied Biosystems, Foster City, CA). Up to 48 libraries were sequenced per lane on a HiSeq 4000 sequencer (Illumina, San Diego, CA) with single-end 100-bp reads. For scRNA-seq, PBMCs from a tumor-bearing dog prior to treatment and from a healthy dog underwent quality control to determine a minimum concentration of 100,000 cells total and sufficient viability over 70%. Single-cell suspension of 700–1200 cells/μl in at least 40 μl of PBS/0.5% BSA suspension buffer was submitted to the UC Davis Genome Center. Library preparation and sequencing using the 10× Chromium Next GEM Single-Cell 3′ version 3.1 Gene Expression protocol were completed by the UC Davis Genome Center.

### Data analysis of 3′ Tag-RNA-Seq and scRNA-seq fastq files

Computational resources were provided by JetStream/XSEDE as a startup allocation. Raw fastq files generated by 3′ Tag-Seq underwent preprocessing via a reproducible pipeline using snakemake. Samples were concatenated, followed by trimming of the first 12 bases and quality trimming using bbduk_qc. Non-rRNA was identified and selected for using bbduk_find_ribo. Reads were then indexed to the reference transcriptome for canines (CanFam3.1), and counts were generated with salmon. Count files generated by salmon were then read into R using the tximport package. Differential gene expression analysis was done using the *DESeq2* package for R, and the Benjamini-Hochberg procedure was used to control false discovery rate. Single-cell fastq files were processed using the *cellranger count* pipeline with generation of and alignment to a *Canis lupus familiaris* reference genome (CanFam3.1) using the *cellranger mkgtf* and *cellranger mkref* pipelines. Further preprocessing was completed in R using the *Seurat* package, and data visualization was completed using R packages *Seurat* and *ggplot2*.

### Ethics statement

The animal study was reviewed and approved by both the University of California, Davis Institutional Animal Care and Use Committee and Clinical Trials Review Board. The title of this approved clinical trial was Allogeneic NK Cells and Palliative Radiotherapy in the Treatment of Canine Cancer (Protocol 21620). Written informed consent was obtained from the owners for the participation of the animals in this study.

## Results

### Canine Ly49 protein structural modeling to murine Ly49 demonstrates overall preserved tertiary structure, particularly the MHC-I binding interface

**FIGURE 1.**
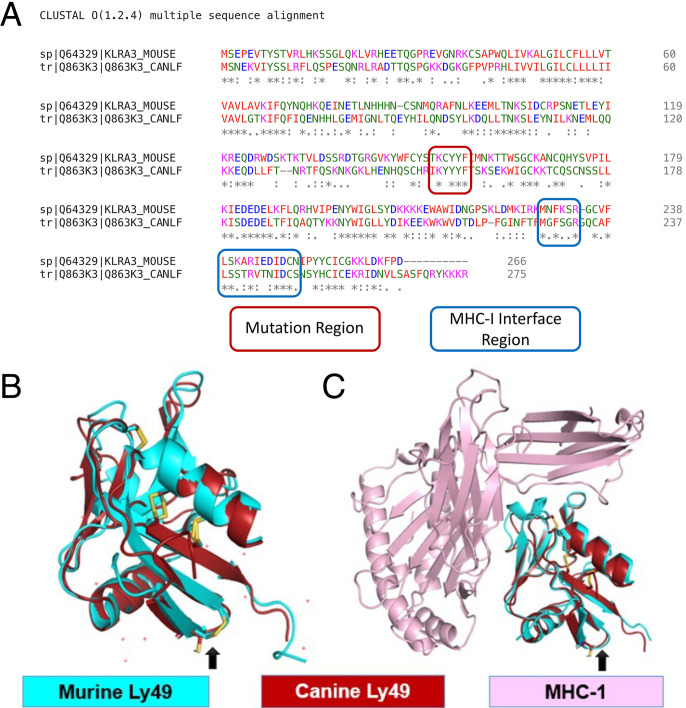
Homology modeling of canine Ly49 compared with murine Ly49. (**A**) BLAST amino acid sequence comparisons for Ly49 between *Mus musculus* (top row) and *Canis familiaris* (bottom row) with the cysteine-to-tyrosine mutation regions (red) and the MHC-I interface region (blue) marked as shown. (**B**) Similarity between overlapping tertiary structures (murine in teal and canine in red) by AlphaFold and ColabFold are shown. Site of cysteine-to-tyrosine mutation is denoted by the black arrow. (**C**) In silico model for binding between murine and canine Ly49 (teal and red) with canine MHC-I (pink). Site of cysteine-to-tyrosine mutation is denoted by the black arrow, which is not predicted to be involved in the Ly49–MHC-I binding interface.

The amino acid BLAST sequences for Ly49 were obtained and analyzed using in silico modeling tools (Fig. 1A). The region in question containing the cysteine-to-tyrosine mutation/polymorphism was identified and found to lie outside of the putative MHC-I interface region. Structural models for the extracellular portion of canine and murine Ly49 were created using AlphaFold2. As seen in the visualization (Fig. 1B), we observed extensive tertiary structural overlap between the two structures. The pairwise root-mean-square deviation between the generated model and chain D in 1P1Z was 0.743 Å. The site of cysteine-to-tyrosine mutation is denoted by the black arrow. This mutation occurs in the context of a secondary structure, a β-pleated sheet, which lends stability to the overall conformation of the tertiary structure despite the loss of the disulfide bond from the cysteine.

Using the structures identified from the protein modeling, protein-protein docking between in silico canine Ly49 and MHC-I was then performed. Fig. 1C depicts the visualization of protein-protein docking between overlapped canine and murine Ly49 and MHC-I. As shown in Fig. 1, the mutation site is not involved in the binding interface. Qualitatively, the overall appearance of canine and murine Ly49 structures while bound to MHC-I are structurally highly similar without loss of structural integrity from the mutation in question.

### Protein-protein docking of canine Ly49 with MHC-I converges to a single low-energy minimum

**FIGURE 2.**
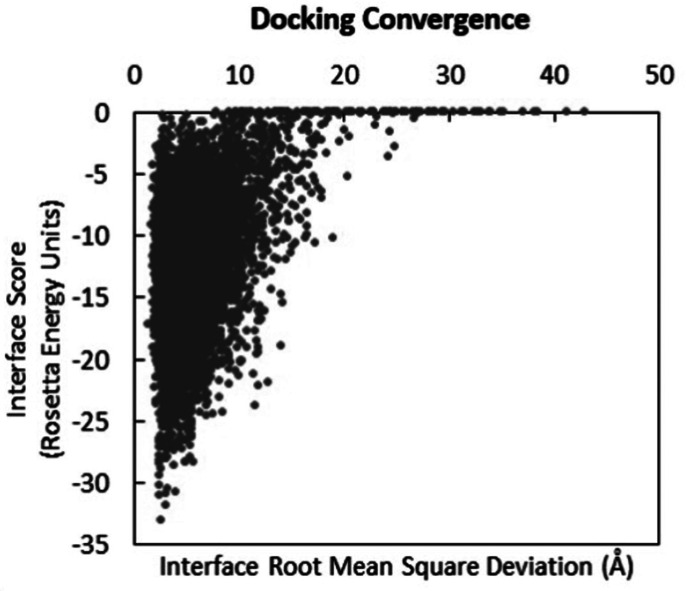
Protein-protein docking of canine Ly49 with MHC-I converges to a single low-energy minimum. Docking convergence between canine Ly49 and MHC-I in Rosetta Energy Units versus the protein-protein interface root-mean-squared deviation depicted. Docking trials are depicted converging at a single low-energy confirmation, the more negative score indicating favorable interactions between Ly49 and MCH-I.

Following the qualitative comparison of overall canine Ly49 structure and predicted intact binding with MHC-I, we then calculated docking convergence between canine Ly49 and canine MHC-I on the basis of the interface score in Rosetta Energy Units versus the protein-protein interface root-mean-squared deviation relative to the input structure. The interface score indicates the (dis)favorability of interactions between the Ly49 and MCH-I, with negative scores being more favorable for binding (31). The interface score is interpreted in the context of interface root-mean-square deviation (measured in angstroms), which was measured relative to the canine system superimposed on the known murine structure. Fig. 2 presents the interface scores of docked canine Ly49 with MHC-I. As depicted on the graph, docking trials converged around a single low-energy conformation, with a highly negative (favorable) interface score. Structures in this minimum diverged from the superimposed input model by 3–5 Å. This result suggests that canine Ly49 is predicted to bind in a single mode with MHC-I and that this mode is similar to that found in the murine system. These simulations provide a preliminary indication that the canine protein is capable of binding MHC-I and could function similarly to the murine protein in complex with MHC-I if validated in functional assays.

### Ly49 is expressed in canine NK cells and demonstrates increased expression following coculture

**FIGURE 3.**
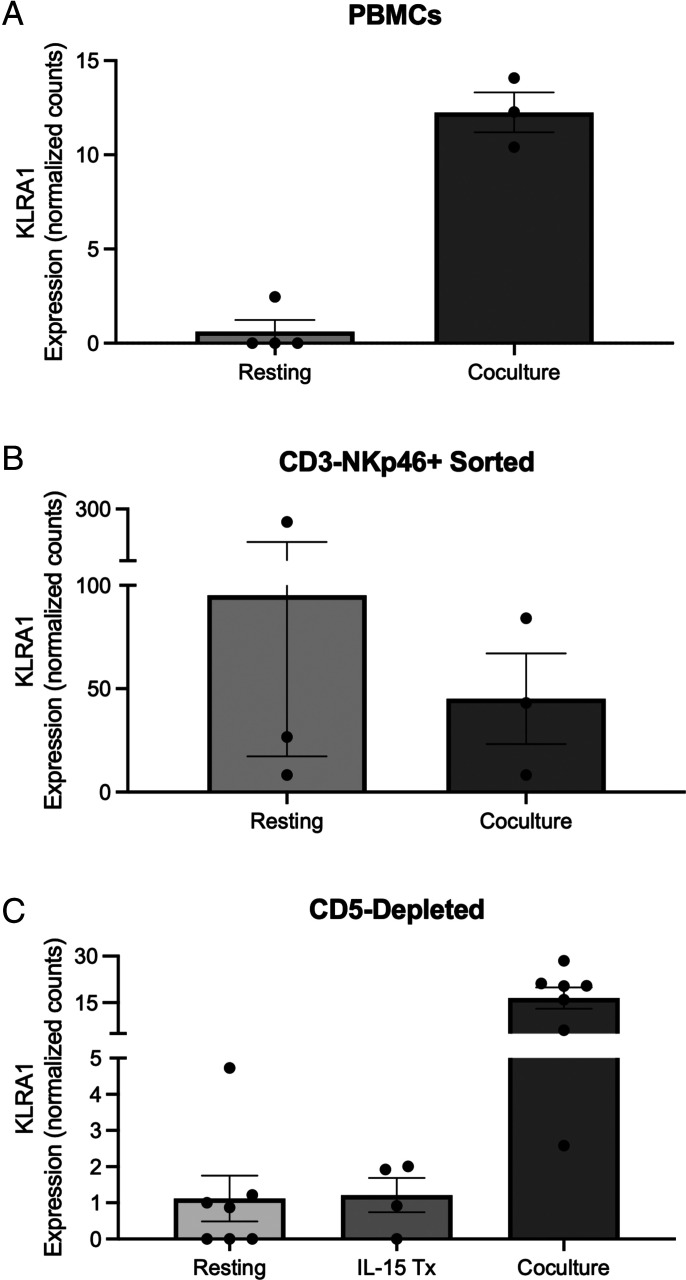
3′ Tag Seq demonstrates expression of Ly49 (KLRA1) across multiple canine donors and conditions. Average KLRA1 expression in canine NK cells expanded from (**A**) bulk PBMCs, (**B**) CD3^−^NKp46^+^ sorted cells, and (**C**) magnetic CD5-depleted cells before and after coculture or IL-15 activation. Graphs display mean values with SEM error bars for each data set.

Because the in silico representations of the canine Ly49 gene demonstrated a favorable conformation for MHC-I docking, we then analyzed in vivo gene expression in canine NK cells. NK cells have been identified by CD5^dim^ expression and can be enriched by magnetic depletion (14). Dog NK cells can also be isolated by flow cytometric analysis and cell sorting, based on NKp46 expression (14). In canines, resting CD3^−^NKp46^+^ populations demonstrate canonical NK markers that are shared among mice and humans (14). However, given the known heterogeneity of NK subsets across species, as well as prior studies identifying a CD5^dim^ subset of canine NK cells, we evaluated both magnetic bead–depleted CD5^dim^ and flow-sorted CD3^−^NKp46^+^ starting populations to investigate Ly49 expression in resting canine NK cells. Additionally, we have found that the coculture of dog PBMCs with irradiated K562 human feeder cells transduced with 4-1BBL (CD137L) and membrane-bound rh-IL21 and low-dose IL-2 results in a highly expanded and activated canine NK population after 14 d (33, 38). Therefore, using this highly purified and activated NK population, we compared RNA expression of canine Ly49 in bulk PBMCs and sorted CD3^−^NKp46^+^ and CD5-depleted NK cells from both steady-state/resting cohorts and cells after coculture with feeder cells and IL-2 for 14 d. Furthermore, we also analyzed CD5-depleted cells following stimulation with IL-15 for an additional comparison of the effect of diverse cytokine stimulation on Ly49 mRNA expression in dog NK cells (39). Differential gene expression between these populations demonstrated that the Ly49 gene, KLRA1, is transcribed by canine NK cells from PBMCs (Fig. 3A) and CD5^dim^ starting populations (Fig. 3B). Furthermore, the expression of KLRA1 is higher in activated than in resting NK cells when starting from bulk PBMCs and CD5-depleted starting populations. Interestingly, KLRA1 expression was highest in flow-sorted CD3^−^NKp46^+^ resting NK cells (Fig. 3B) and decreased following coculture of purified CD3^−^NKp46^+^ NK cells with irradiated feeder cells, although this decrease in KLRA1 expression was not statistically significant.

### Single-cell analysis of canine Ly49 expression

**FIGURE 4.**
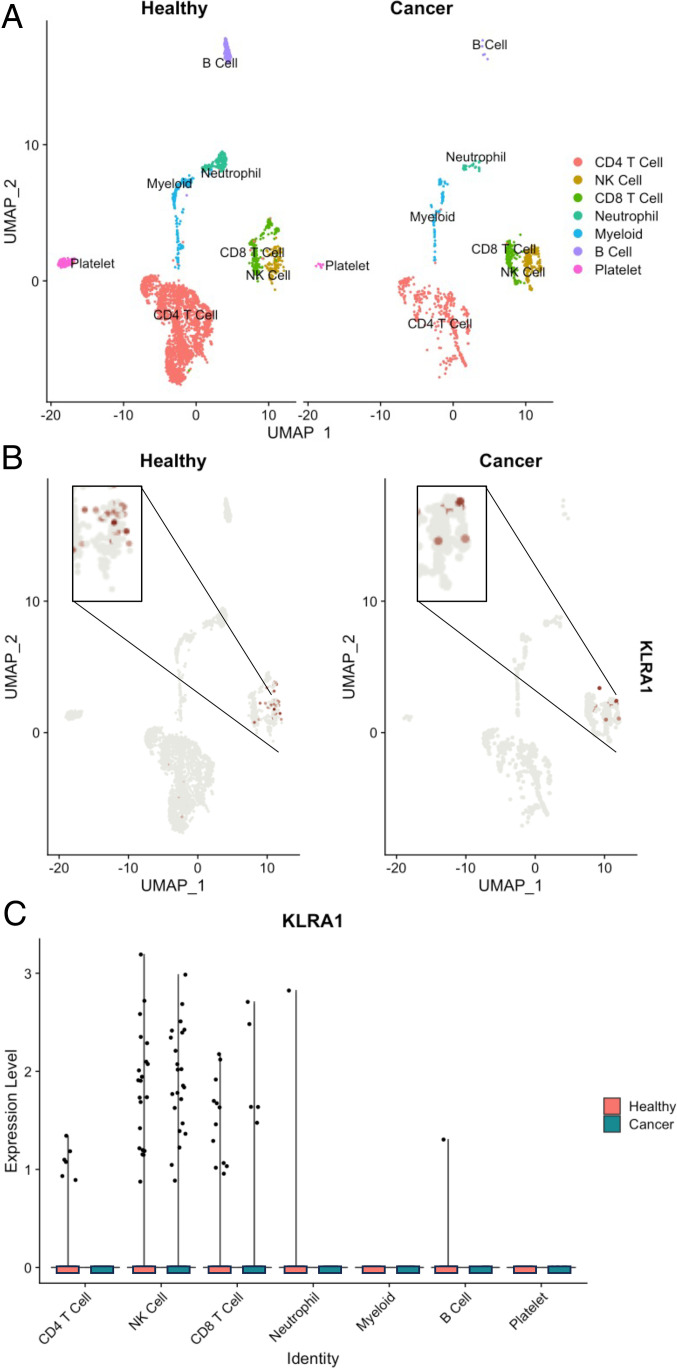
scRNA-seq analysis illustrating KLRA1 expression in canine NK cells in healthy and cancer-bearing donors and association with NK-related genes. Uniform manifold approximation and projection (UMAP) plots of an integrated dataset of PBMC samples from a healthy donor and a cancer-bearing donor prior to treatment (**A**) showed a distinct NK cell population with (**B** and **C**) KLRA1 expression limited largely to the NK cell cluster.

To further explore the in vivo gene expression Ly49^+^ and Ly49^−^ subsets, we performed scRNA-seq on PBMCs obtained from both healthy beagle and a tumor-bearing donors. Cell-type–specific differentially expressed genes identified the NK cell cluster in an integrated dataset of PBMCs from both dogs, including GZMB, NCR3/NKp30, GZMA, and IL12RB2 (log_2_ fold change >2, *p*_adj_ < 0.001) (Fig. 4A). When examining gene expression levels across all cells, expression of KLRA1/Ly49 was predominantly restricted to the NK cluster in both samples (Fig. 4B, 4C), although we observed some expression in CD8^+^ T cells. Taken together, these data were consistent with our hypothesis that Ly49 is an important, potentially functional protein for canine NK cells.

### Spectral clustering of canine Ly49 expression

**FIGURE 5.**
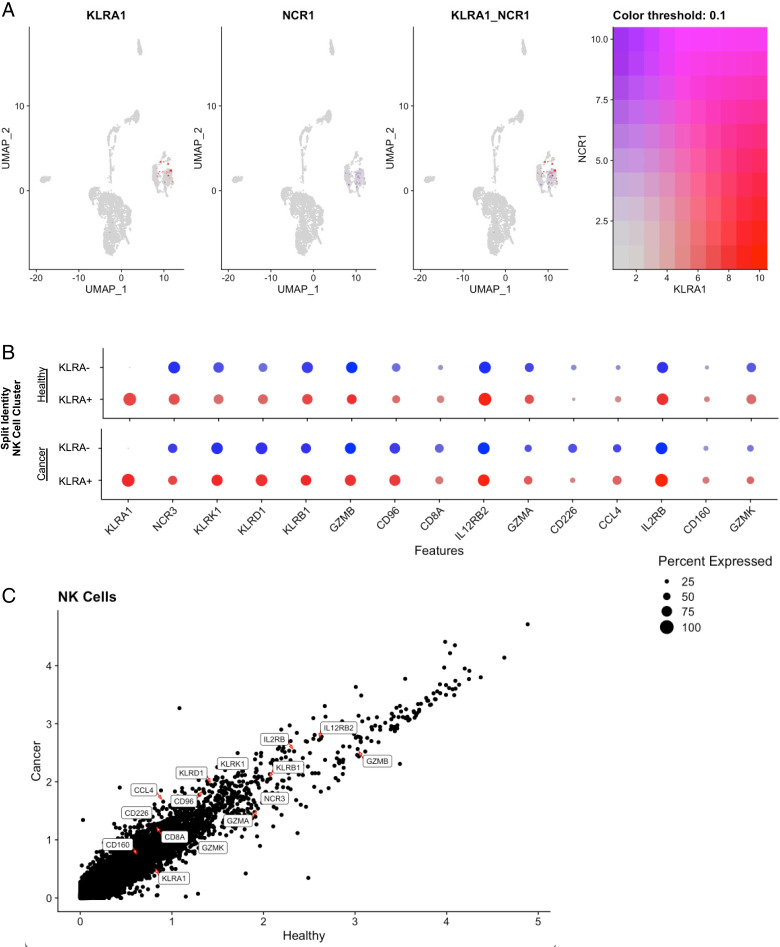
scRNA-seq analysis displays association between KLRA1 and NK-related genes. (**A**) Uniform manifold approximation and projection (UMAP) demonstrating KLRA1 coexpression with NCR1/NKp46 in the NK cell cluster. (**B**) KLRA^+^ and KLRA^−^ cells in the NK cell cluster of the healthy and cancer samples were subset using a threshold of 1, and the percentage of cells expressing NK-related genes was visualized by dot plot. (**C**) NK-related genes are labeled in a scatterplot of the average expression of NK cell populations, showing no significant differences between healthy and cancer samples.

To better understand the context of KLRA1 expression, we then evaluated the coexpression of KLRA1 and NK-related genes in an integrated analysis of data from both the healthy and cancer-bearing PBMC subjects. First, we observed strong concordance between the expression of NCR1/NKp46 and KLRA1 expression in cells within the NK cluster (Fig. 5A). However, when comparing KLRA1^+^ and KLRA1^−^ cells within the NK cluster, we observed no significant differences in coexpression of other NK or activation markers (Fig. 5B), suggesting that KLRA1 does not significantly alter NK phenotype, at least at the gene expression level. Additionally, a dot plot of average gene expression showed no significant change in KLRA1 or related NK gene expression when comparing single-cell analysis of NK cells from cancer-bearing and healthy donor dog PBMCs, suggesting that the presence or absence of cancer has little immunoregulatory effect on KLRA1 expression in canine NK cells. Overall, these sequencing data further support KLRA1 as actively transcribed within canine NK cells in multiple and distinct states.

## Discussion

Prior studies suggesting that KIRs are pseudogenes and that the Ly49 protein is also nonfunctional in dogs on the basis of specific amino acid changes has left a major gap in the literature in terms of how dog NK cells recognize MHC-I (21, 24). Our study aimed to fill this gap by using protein structure modeling, protein-protein docking, and RNA-seq data to better understand the impact of the cysteine-to-tyrosine conversion at position 168 in dog Ly49 and its impact on MHC-I binding. For the first time, using these complementary experimental techniques, we show that the cysteine-to-tyrosine mutation in question does not appear to significantly alter the structural conformation of canine Ly49, though additional functional data would be required to confirm the computationally determined binding modality. In addition, we also discovered that this mutation, which has led investigators to previously characterize canine Ly49 as nonfunctional (21), is not located at the predicted binding interface, supporting our inference that canine Ly49 appears to bind MHC-I in the same conformation as murine Ly49 with favorable energy kinetics. Finally, our sequencing data provide additional evidence that canine Ly49 is transcribed by canine NK cells in the resting and activated states, further reinforcing our hypothesis that Ly49 is potentially a functional protein in canine NK cells with important implications for NK biology in dogs.

NK cells have evolved to fill an important niche bridging innate and adaptive immunity, where they are critical to immune regulation, pathogen clearance, and cancer surveillance. Detailed studies have uncovered key mechanistic insights into NK biology in mice and humans, including critical concepts such as the missing self hypothesis, NK education and licensing, maturation, and memory formation (40–43). However, key species differences between mouse and human NK cells have hindered clinical translation. These key differences include conventional phenotypic markers for NK identification (CD56 human, NK1.1/NKp46, CD49b mouse); expression of cytolytic markers in the resting versus activated state (perforin and granzyme); stages and tissues of maturation; and, importantly, mechanisms of MHC-I recognition (43–45). MHC-I recognition is fundamental to NK immunoregulation, and, unlike major constituents of the immune system such as T cells and B cells, where Ag-specific activating signals are delivered in the context of MHC-I presentation, MHC-I binding stereotypically delivers a strong inhibitory signal to NK cells that can be flipped to activation, especially for licensed NK cells, via MHC-I downregulation and loss of “self.” In the mouse, NK cells recognize MHC-I via Ly49 alleles, whereas in humans, receptor-ligand binding occurs via KIRs.

Previously, a consensus was formed, based largely on studies by Gagnier et al. and Hammond et al., that the mechanism of MHC-I recognition in dogs was unknown (21, 24). Hammond et al. found that KIR genes diverged into two lineages, 3DL and 3DX, with Carnivora species, such as dogs and pinnipeds, having a KIR gene of the 3DL lineage between genes LILR and *FCAR* (24). In dogs, short-read DNA sequencing showed a missing KIR gene with the adjacent FCAR gene being truncated, leading to the hypothesis that the KIR gene was deleted in dogs (24). Prior to this, Gagnier et al. separately observed an Ly49 gene in the canine genome using Southern blot technology. However, although they determined that the coding region of the Ly49 gene was intact in dogs, the authors concluded that the protein was nonfunctional (21), based on the absence of highly conserved cysteine residues within the C-type lectin-like domain that were present in other species. These cysteine residues were believed to be critical for functional Ly49 genes, based on protein structure. Because the cysteine at position 168 has been replaced with tyrosine in the dogs, the authors questioned whether it would have the same conformation and therefore the same functionality (21). Cysteine residues form disulfide bonds that supply stability to protein structures and influence conformational changes necessary for function. Data from our study confirm the presence of this mutation, but computational modeling suggests it does not interfere with binding because the mutation exists within a stable β-pleated sheet secondary structure. Importantly, this mutation is located separately from the putative Ly49–MHC-I binding site.

Structural modeling of canine Ly49 displays a tertiary structure with virtually complete overlap with murine Ly49, which is known to be functional, and locates the mutation as separate from the binding site. This unobstructed binding to MHC-I suggested by our structural modeling is further substantiated by protein-protein docking convergence of canine Ly49 and MHC-I at the binding site. The two proteins dock together with a single low-energy minimum on convergence, signifying a favorable atomic structure. Together, these data suggest that canine Ly49 is likely capable of binding to MHC-I. Although our computational modeling data suggest that binding can still occur, it is conceivable that this mutation may have a yet unidentified effect because there are reports of similar mutations inactivating gene function in other settings (46). We then performed RNA-seq and differential gene expression analysis on resting and activated canine NK cells to indirectly assess for gene function through mRNA expression. Using multiple conditions and techniques to assess resting and activated canine NK cells, we observed robust expression of Ly49 RNA transcripts almost exclusively in NK cell subsets, leading us to propose that the mutation does not hinder gene expression.

The finding that dogs transcribe Ly49 is consistent with other literature showing upregulation of Ly49 in dog NK cells in different contexts. Interestingly, other studies, in addition to our own, show that Ly49 is upregulated in activated NK cells (47–49), contradicting the idea of Ly49 functioning through an inhibitory signal. Dog NK cells had a 295-fold increase in Ly49 mRNA expression after 21-d culture and increased activity compared with PBMCs, even greater than the 32.4-fold increase in the activating receptor, NKG2D (47). Similarly, a 14-d expansion of dog cytotoxic large granular lymphocytes increased Ly49 mRNA expression significantly (25.5-fold) compared with baseline PBMCs (48). Collectively, these results prompt the question whether canine Ly49 may be at least partially activating rather than strictly inhibitory as previously thought. This concept has also been proposed in mice, which have 8–21 Ly49 genes with varying functions, in contrast to the 1 Ly49 gene in dogs (50). For example, murine Ly49A, Ly49C, and Ly49G are inhibitory, whereas Ly49D and Ly49H are activating, at least in C57BL/6 mice (51). Further studies are needed to determine whether canine Ly49 is activating or inhibitory and whether similar Ly49 or KIR-like genes exist with contrasting or redundant function.

Although this study provides critical insight on one possible mechanism by which canine NK cells can successfully bind and recognize MHC-I, it does not establish that there is only a single mechanism. Our study is further limited by an inability to demonstrate definitive functional implications of Ly49 gene expression in dog NK cells. The fact that a single Ly49 gene has been observed in dogs does not exclude the existence of similar levels of Ly49 polymorphisms characteristically observed in mice, and studies have suggested that a second canine Ly49 gene could be present in the genome (21, 48). It is possible that previous and current techniques are not capable of identifying Ly49 genes in dogs that have characteristics distinct from the known sequence (21). Given the presence of multiple Ly49 receptors in mice with significant genetic polymorphisms and strain heterogeneity, it is notable that we have observed evidence of only one Ly49 receptor in dogs at this time. This may represent a knowledge gap because similar heterogeneity and polymorphisms would be expected to be conserved across mammalian species. However, additional mechanisms unique to canines may also account for the lack of Ly49 polymorphism seen, because dogs have been observed to have differential viral drivers of their immune repertoire, and the reported lack of CMV infection in dogs may underlie unique NK biology and function, including Ly49 receptor expression (52). Humans are similar to dogs in that they have only one known Ly49-like gene, Ly49L, but it has a premature stop codon that does not contain part of a C-terminal lectin domain, providing more clear evidence that it is nonfunctional (53). Because modern sequencing and computational methods have not yet been directed toward interrogating the canine KIR gene, dogs may also potentially have an undiscovered KIR gene.

Data from baboon lymphocytes have demonstrated that functional Ly49 and KIR gene expression may be present simultaneously, thereby providing an evolutionary map in which Ly49 genes diverged and were selected for in mice but became inactive or low copy in other species (54). An additional hypothesis proposes that heterogeneous NK populations may have varying receptors for MHC-I recognition. For example, CD56^bright^ human NK cells express NKG2A without KIR and can be hyporesponsive, whereas CD56^dim^ cells express KIR with or without NKG2A and show greater cytolytic activity (41, 55, 56). Similarly, diverse canine NK cell populations have been identified, and interrogation of these distinct NK cell populations may reveal additional complexity of MHC-I recognition and NK regulation among heterogeneous NK subsets. These follow-up studies will provide important additional characterization of NK biology in dogs and how they are relevant for cross-species studies.

Ultimately, a better understanding of how canine NK cells recognize MHC-I is needed because NK cell interactions with MHC-I are critical to self-tolerance and licensing/education (41, 57). Although future studies such as discovery-based proteomics with mass spectrometry are needed to better define the structure and function of canine Ly49 protein, the present study makes critical progress in resolving canine NK/MHC-I recognition strategies because we uncover significant evidence that canine NK cells uniquely express Ly49 transcripts and that canine Ly49 is expected to be capable of binding to MHC-I. These data can form the baseline for future studies designed to understand canine NK cells and their role in shaping antiviral and antitumor responses in dogs.
